# Type I interferon rapidly restricts infectious hepatitis C virus particle genesis

**DOI:** 10.1002/hep.27333

**Published:** 2014-10-27

**Authors:** Luke W Meredith, Michelle J Farquhar, Alexander W Tarr, Jane A McKeating

**Affiliations:** 1 Viral Hepatitis Research Group, Centre for Human Virology, University of BirminghamBirmingham, UK; 2 School of Molecular Medical Sciences and the Nottingham Digestive Diseases Centre Biomedical Research Unit, University of Nottingham, Queen's Medical CentreNottingham, UK; 3 NIHR Liver Biomedical Research Unit, University of BirminghamBirmingham, UK

## Abstract

Interferon-alpha (IFNα) has been used to treat chronic hepatitis C virus (HCV) infection for over 20 years with varying efficacy, depending on the infecting viral genotype. The mechanism of action of IFNα is not fully understood, but is thought to target multiple stages of the HCV lifecycle, inhibiting viral transcription and translation leading to a degradation of viral RNA and protein expression in the infected cell. IFNα induces the expression of an array of interferon-stimulated genes within minutes of receptor engagement; however, the impact of these early responses on the viral lifecycle are unknown. We demonstrate that IFNα inhibits the genesis of infectious extracellular HCV particles within 2 hours of treating infected cells, with minimal effect on the intracellular viral burden. Importantly, this short duration of IFNα treatment of infected cells significantly reduced cell-free and cell-to-cell dissemination. The secreted viral particles showed no apparent change in protein content or density, demonstrating that IFNα inhibits particle infectivity but not secretion rates. To investigate whether particles released from IFNα-treated cells have a reduced capacity to establish infection we used HCV lentiviral pseudotypes (HCVpp) and demonstrated a defect in cell entry. Using a panel of monoclonal antibodies targeting the E2 glycoprotein, we demonstrate that IFNα alters glycoprotein conformation and receptor utilization. *Conclusion*: These observations show a previously unreported and rapid effect of IFNα on HCV particle infectivity that inhibits *de novo* infection events. Evasion of this response may be a contributing factor in whether a patient achieves early or rapid virological response, a key indicator of progression to sustained virological response or clearance of viral infection. (Hepatology 2014;60:1890–1900)

Hepatitis C virus (HCV) infection is a major cause of chronic liver disease that affects 170 million people worldwide, leading to cirrhosis and hepatocellular carcinoma. For many years the only available therapy was pegylated type I interferon alpha (IFNα) and ribavirin that cured less than 50% of cases in a genotype-dependent manner, prompting the development of direct-acting antiviral agents (DAAs) targeting the viral replicase.^1^ The approval of HCV protease and polymerase inhibitors and combination treatment with IFN has led to a significant increase in patient response rates, providing a new standard of care.^2^

Type I IFN stimulates the expression of a number of interferon-stimulated genes (ISGs) that inhibit HCV genome translation and replication^3^,^4^ and protect neighboring uninfected cells from viral infection. Once IFN pathways are activated the downstream response has enormous breadth, as noted by the many hundreds of ISGs. In a recent screen 380 ISGs were tested for their ability to inhibit genotype 2a HCV replication and at least 25 genes were identified that reduced viral replication.^5^ Zhao et al.^6^ reported that silencing 60 ISGs prior to IFN treatment revealed no individual gene knockdown that could rescue the inhibitory effect of IFN on HCV replication. This finding is consistent with the widely accepted hypothesis that the IFN system works in a combinatorial fashion, with multiple ISGs contributing to antiviral responses with no single ISG serving as a “magic bullet.”

Mathematical modeling of HCV replication kinetics following treatment has provided valuable insights into the mechanism of action of antiviral agents.^7^,^8^ HCV RNA declines in a biphasic pattern after IFN therapy, with the initial phase representing a decrease in viral production from infected cells, while the slower and more variable phase is attributed to the loss of infected cells by way of the stimulated immune system. Importantly, there are multiple outcomes observed following IFN therapy, including rapid, partial, and nonresponse, where a rapid virological response is frequently associated with a cure even more than the commonly used IL-28B polymorphisms.^9^ Recent modeling studies suggest that for maximal efficacy IFN needs to inhibit infectious virus production and *de novo* transmission events.^10^,^11^ However, the majority of reports studying the impact of IFN on HCV replication have not analyzed these steps in the viral lifecycle.

Reports of IFN-mediated signaling generally study cellular responses within minutes of receptor engagement. However, studies investigating the antiviral effects of IFN measure HCV RNA and proteins several days posttreatment. We demonstrate that treating HCV-infected Huh-7 cells with IFNα for 1 hour reduces the infectivity of extracellular particles with no detectable effect on particle secretion. We failed to detect soluble antiviral effectors released from IFN-treated cells and purified particles show reduced infectivity, demonstrating a direct effect of IFNα on extracellular HCV particles. Studies with lentiviral HCV pseudoparticles (HCVpp) demonstrate that IFN alters E2 glycoprotein conformation that limits receptor-dependent internalization. This is the first report of such a rapid and direct effect of IFNα to reduce particle specific infectivity in the absence of any detectable impact on viral RNA load, raising questions on the interpretation of early phase IFN responses that are solely based on viral RNA measurements.

## Materials and Methods

### Cell Lines and Antibodies

Huh-7 or Huh-7.5 cells were propagated in Dulbecco's modified Eagle's medium (DMEM) / 10% fetal bovine serum and 1% nonessential amino acids. Anti-CD81 2s131 and anti-SR-BI (gift from Pfizer) were previously reported.^30^ A panel of rodent^31^ and human monoclonal antibodies (mAbs) mapping to linear or conformation-dependent HCV E2 epitopes were previously reported.^32^,^33^ Immunoglobulin was isolated from the sera of normal or chronically infected HCV patients by protein G-conjugated Sepharose beads as previously reported.^12^

### ISG Analysis

HCV-infected cells were treated with IFNα and cellular RNA extracted using RNAeasy Miniprep Kits (Qiagen). ISG expression was quantified using the Human Interferon Response PCR Array and analyzed using RT^2^ Profiler Analysis software (SABiosciences).

### Sodium Dodecyl Sulfate-Polyacrylamide Gel Electrophoresis (SDS-PAGE) and Western Blotting

Cells were harvested in lysis buffer containing protease inhibitors, clarified by centrifugation (20,000*g*, 10 minutes), and protein concentration quantified by BCA (Pierce). Normalized protein lysates or virus pellets resuspended in Laemmli buffer were separated by SDS-PAGE, transferred to PVDF membranes, and probed for HCV-E2 (mAb 3/11) or HCV-Core (mAb C750, Thermo Scientific) and expression quantified by enhanced chemiluminescence and densitometric analysis using ImageJ software.

### HCVcc Infectivity, Density Analysis, and Transmission Assay

Plasmids encoding HCV SA13/JFH, J6/JFH, or H77/JFH were used to generate RNA and electroporated into Huh-7.5 cells.^12^ IFNα was added to cells for defined time periods followed by thorough washing and harvesting of extracellular media. Infected cells were fixed with paraformaldehyde, stained for viral antigen expression with mAbs specific for NS5A (9E10), Core (C750), or E2 glycoprotein (9/75, 3/11, 6/53) and isotype-matched Alexa-488 conjugated IgG. Viral antigen expressing cells were enumerated by fluorescent-activated cell sorter (FACS) and protein expression assessed by measuring mean fluorescent intensity (MFI). Extracellular media was collected, concentrated through 100,000 MWCO concentrators (Sartorius), loaded onto 10-60% Iodixanol gradients, and centrifuged at 100,000*g* for 16 hours at 4°C. Fractions were collected and density assessed by weight, infectivity by inoculating naïve Huh-7.5 cells, and HCV RNA by quantitative reverse-transcription polymerase chain reaction (qRT-PCR).

HCV-infected cells were labeled with fluorescent cell-tracker CMFDA and cocultured with an equal number of naïve Huh-7 cells at 2.5 × 10^4^ cells/cm^2^. Extracellular virus is neutralized with anti-HCV Ig or control Ig (150 μg/mL) and transmission stopped at indicated times by anti-CD81 (2s131, 10 μg/mL).^12^ Cells were harvested and culture media analyzed for infectious virus, expressed as the number of focus-forming units (FFU)/mL. Newly infected target cells were identified by staining for HCV NS5A and quantified by flow cytometry.

### HCVpp Infectivity, Glycoprotein Conformation, and Neutralization

Luciferase reporter pseudoparticles expressing HCV envelope glycoproteins (HCVpp), or no-glycoprotein controls, were generated in 293T cells as previously reported.^31^ HCVpp-293T producer cells were treated with IFNα for 1 hour, unbound IFN removed, and the cells incubated for 1 hour prior to collecting extracellular media to measure infectious HCVpp and staining cells for E2 expression and quantification by flow cytometry. All anti-E2 mAbs were used at subsaturating concentrations. HCVpp stocks were incubated with anti-E2 mAbs or polyclonal anti-HCV Ig for 1 hour prior to infecting Huh-7.5 cells. Receptor neutralization was performed by adding anti-SR-BI, anti-CD81, or isotype control antibody to Huh-7.5 cells for 1 hour prior to infection.

HCV E2 expression in p24 normalized HCVpp containing supernatants was determined by enzyme-linked immunosorbent assay (ELISA). Briefly, Immulon plates (Thermo) were coated with GNA (Sigma, UK) and empigen-lysed HCVpp preparations added for 2 hours at room temperature. Bound E2 was detected with a panel of anti-E2 antibodies and HRP-conjugated anti-rat Ig. For HIV-1 p24 quantification, MaxiSorp plates (Nalge Nunc) coated with anti-p24 antibody D7324 were used to capture HCVpp (0.5% Tween-20 / 0.1% casein / 1.44M NaCl / 250 mM Tris-Cl, pH 7.5) and bound antigen detected with biotinylated rabbit anti-p24 and streptavidin-HRP (BD Pharmingen). Bound HRP-conjugates were quantified colorimetrically after reaction with a TMB substrate (BioFX).

## Results

### IFNα Rapidly Induces an Antiviral State

**Fig 1:**
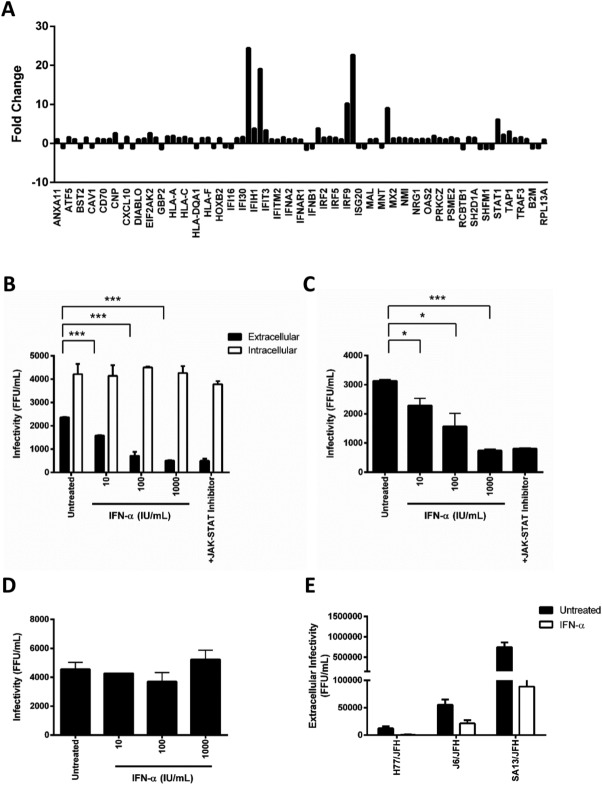
IFNα rapidly reduces extracellular virus infectivity. HCV (SA13/JFH)-infected Huh-7.5 cells were treated with IFNα (1,000 IU/mL) for 1 hour, cellular RNA extracted, and analyzed in a selected mRNA IFN PCR array. Results show the fold regulation of 64 ISGs in infected cells compared to untreated (A). HCV strain SA13/JFH-infected Huh-7.5 cells were treated with an increasing dose of IFNα for 1 hour, washed thoroughly, incubated for a further 1 hour prior to collecting extracellular media and cellular lysates to measure infectious virus by inoculating naïve Huh-7/5 targets. Huh-7.5 targets were treated with Jak/Stat inhibitor (InSolution JAK Inhibitor 1, 10 μM) for 1 hour prior to infecting with virus harvested from cells treated with the highest dose of IFNα (B). Virus was purified from the extracellular media by ultracentrifugation through 20% sucrose, infectivity measured in the presence or absence of Jak/Stat inhibitor (C) and the antiviral activity of the clarified supernatant assessed by pretreating Huh-7.5 cells for 2 hours prior to infecting with HCVcc (D). The effect of IFNα (1,000 IU/mL, 1-hour treatment) on extracellular infectious virus released from H77/JFH, J6/JFH, and SA13/JFH infected Huh-7.5 cells (E). Results shown are the mean and standard deviation of three experiments, and significance calculated using Student *t* tests (*P < 0.05, **P < 0.01, ***P < 0.005) comparing treated sample to untreated controls.

While the clinical impact of IFN therapy on HCV replication kinetics has been extensively studied, our understanding of early IFN-signaling events in the virus lifecycle is limited. Using a commercial IFN-selected PCR array, we confirmed a significant induction of multiple ISGs within 1 hour of treating HCV-infected Huh-7 cells with IFNα (Fig. 1A). To assess whether this early response has an impact on virus replication, infected cells were treated with varying doses of IFNα for 1 hour and virus infectivity measured. The treated cells were washed extensively to remove exogenous IFNα prior to harvesting samples to ensure minimal carryover when measuring viral infectivity. We noted a dose-dependent decrease in extracellular infectivity with no observable effect on the intracellular pool of infectious virus (Fig. 1B). Importantly, the infectivity of extracellular virus harvested from IFN-treated cells was not restored by pretreating Huh-7 cells with Jak/Stat inhibitor (InSolution JAK Inhibitor 1) (Fig. 1B). Extracellular media from IFN-treated Huh-7 cells had no detectable antiviral activity, suggesting a lack of soluble antiviral factors. To investigate whether IFN affects particle infectivity, we purified the extracellular virus by ultracentrifugation and demonstrated a significant reduction in the infectivity of particles (Fig. 1C). In parallel, the supernatant from ultracentrifuged IFN-treated samples had no effect on HCV infection of naïve Huh-7.5 cells (Fig. 1D), demonstrating that IFN reduces HCV particle infectivity. Finally, we demonstrated that IFN reduces the level of extracellular infectious virus of chimeric strains expressing genotype 1 (H77), genotype 2 (J6), or genotype 5 (SA13) structural proteins (Fig. 1E).

**Fig 2:**
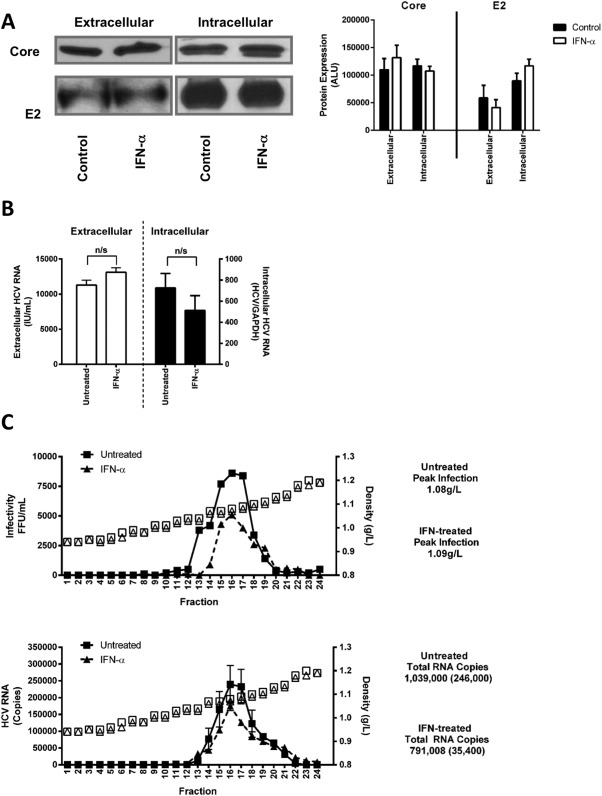
IFNα does not perturb HCV particle composition or density. HCV (SA13/JFH)-infected Huh-7.5 cells were treated with IFNα (1,000 IU/mL) for 1 hour, washed thoroughly, and the extracellular media collected for a further 1 hour. HCV Core and E2 expression in extracellular purified virus and cell lysates was assessed by western blot and densitometric imaging (expressed in arbitrary light units, ALU) (A) while HCV RNA was quantified by qRT-PCR (B). The lipid density of the treated and untreated virus was assessed using 10-60% Iodixanol gradient centrifugation: the graphs depict the infectivity, HCV RNA, and density of each fraction from IFN-treated or untreated cells (C).

The observation that IFNα reduces extracellular HCV infectivity could be explained by the reduced secretion of particles and/or a loss in particle infectivity. To determine whether IFNα affects HCV particle secretion we measured viral-encoded core, envelope glycoprotein E2, and genomic RNA levels in infected cells and in purified extracellular particles. This short-term IFNα treatment had no detectable effect on intracellular or extracellular particulate viral protein (Fig. 2A) or RNA expression (Fig. 2B), suggesting that IFNα has minimal effects on virus secretion. HCV assembly is dependent on the VLDL pathway and extracellular lipoviral particle infectivity has been reported to associate with particle density. IFNα had minimal impact on HCV particle density, with untreated particles showing a peak of infectious virus at a density of 1.09 g/L compared to 1.08 g/L for IFNα-treated cells and no significant change in viral RNA levels (Fig. 2C). In summary, these results show that IFNα reduces the infectivity of extracellular HCV particle while having minimal impact on secretion or particle density.

### IFNα Rapidly Prevents *De Novo* HCV Infection

To investigate the impact of this rapid IFN-mediated response on infectious particle genesis on viral dissemination, we used a recently developed single-cycle infection assay that quantifies *de novo* infection events.^12^ This assay uses the neutralizing capacity of anti-HCV Ig to limit extracellular virus infectivity to discriminate between cell-free and cell-to-cell infection events. Thus, we were able to study the temporal effect(s) of IFNα on HCV-infected cells and to discriminate from bystander effects on naïve cells. HCV SA13/JFH infected Huh-7.5 cells were treated for 1 hour with IFNα (1,000 IU/mL), washed thoroughly to remove exogenous IFN, and labeled with CMFDA prior to coculturing with naïve Huh-7.5 cells in the presence or absence of neutralizing anti-HCV Ig. At indicated timepoints, viral dissemination was stopped by adding an antibody targeting the CD81 receptor (2s131, 10 μg/mL), then incubated for a further 24 hours prior to harvesting cells for flow cytometric detection of newly infected target cells (NS5A^+^/CMFDA^-^) and collection of extracellular media to measure virus infectivity.

**Fig 3:**
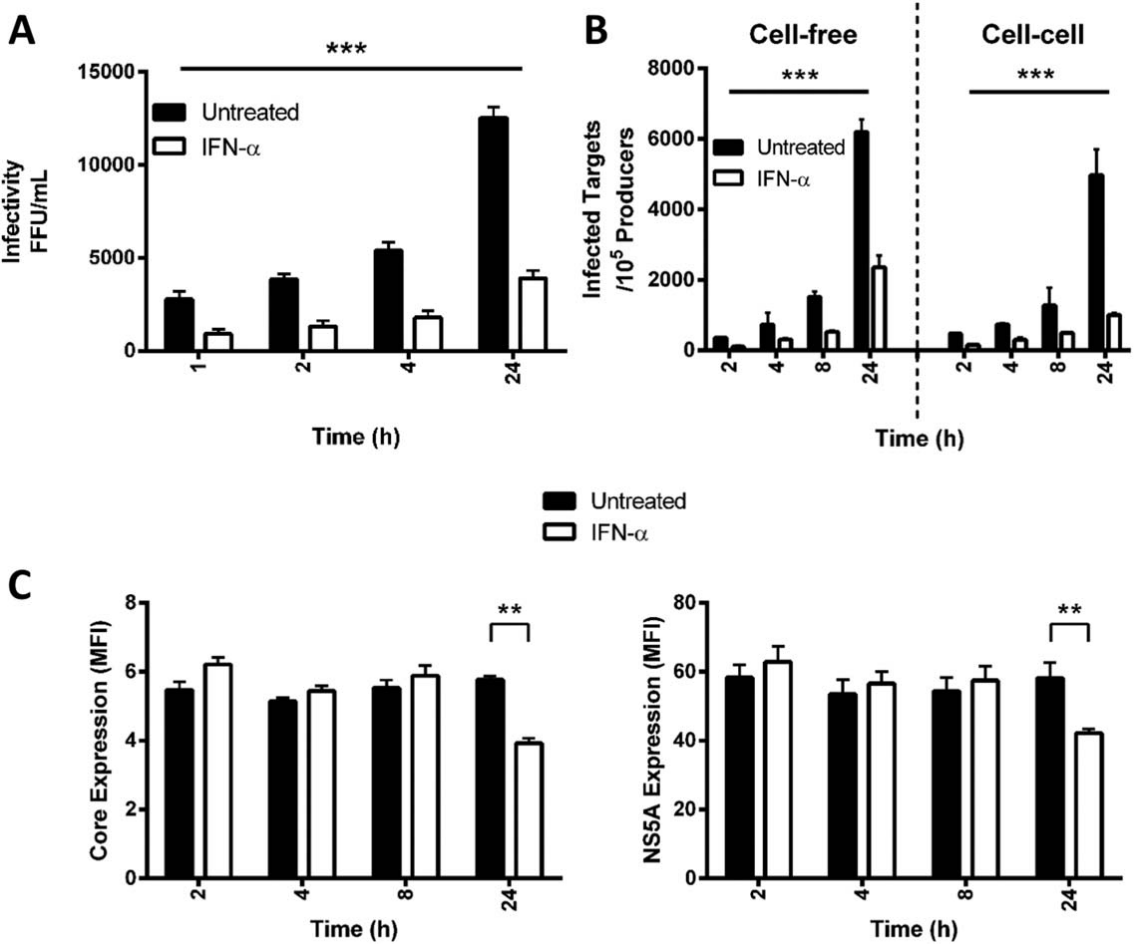
IFNα inhibits cell-free and cell-to-cell HCV transmission. HCV (SA13/JFH)-infected Huh-7.5 cells were treated with IFNα (1,000 IU/mL) for 1 hour, washed thoroughly to remove IFNα, labeled with CMFDA, and cocultured with naïve Huh-7.5 cells. One hour post-coculture anti-HCV Ig was added to neutralize extracellular virus and at indicated timepoints anti-CD81 was added to block all subsequent transmission events. Twenty-four hours post-coculture the levels of the infectious virus in the extracellular media were measured (A) and *de novo* infection events determined by flow cytometry (B). The results represent the mean and standard deviation of three experiments, and significance calculated using Student *t* tests (*P < 0.05, **P < 0.01, ***P < 0.005) comparing treated sample to untreated controls. To assess whether IFNα induced any viral protein degradation during this time period, CMFDA-labeled infected cells were stained for HCV Core or NS5A (C) and the data presented as mean fluorescent intensity (MFI).

As expected, we noted significant reductions in the level of extracellular infectious virus in the IFN-treated samples at all timepoints (Fig. 3A). IFNα treatment of infected cells for 1 hour significantly reduced cell-free and cell-to-cell dissemination routes within the first 2 hours of coculture and this effect was maintained for the duration of the assay (Fig. 3B). We noted a minimal effect of IFNα on the expression of HCV Core or NS5A in infected cells within the first 8 hours following treatment (Fig. 3C). These observations highlight a new role for IFN to limit the genesis of infectious HCV particles and inhibit *de novo* transmission events.

### IFNα Perturbs HCV Glycoprotein-Dependent Particle Infectivity

**Fig 4:**
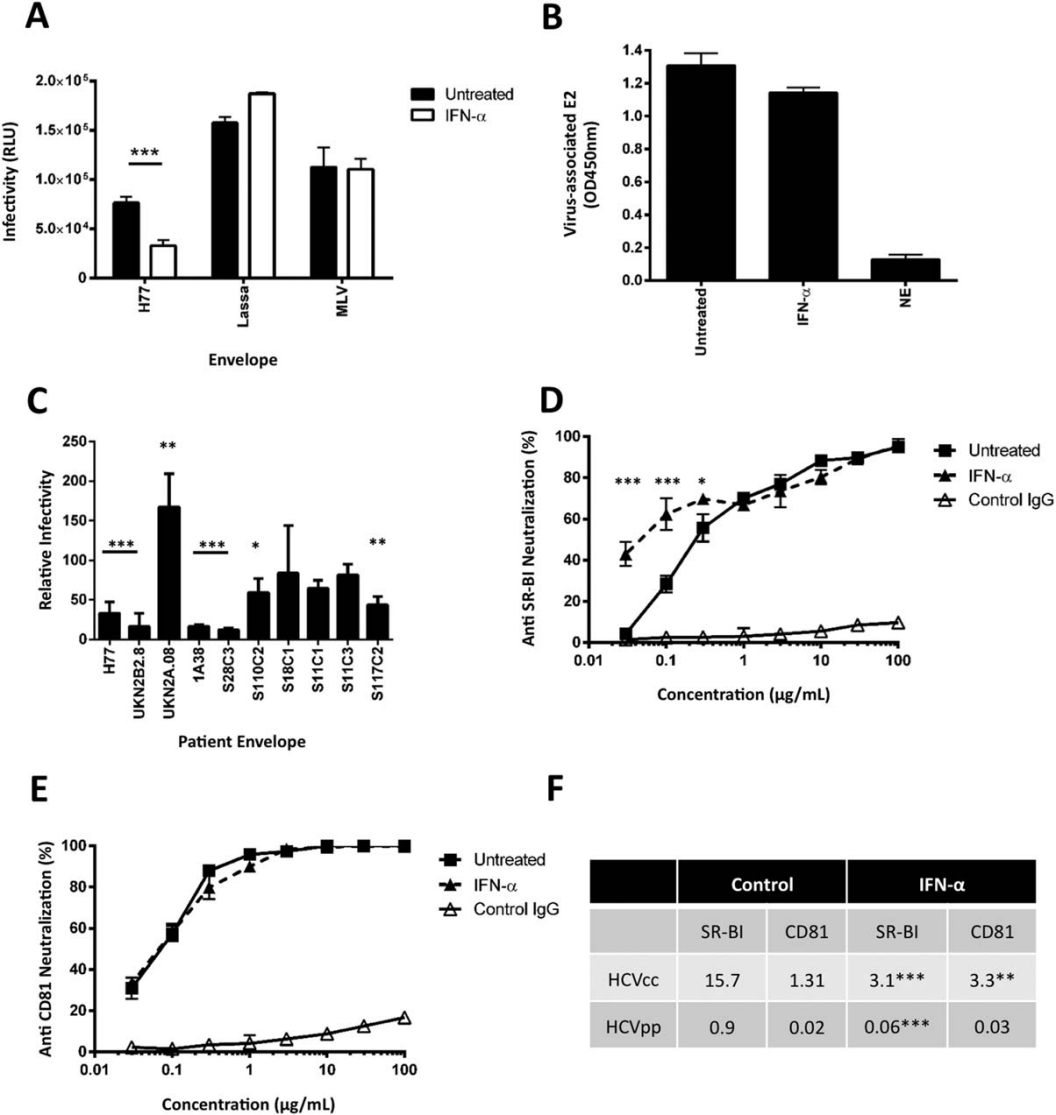
IFNα-treated HCVpp show defective entry and increased sensitivity to anti-SR-BI neutralization. 293T cells expressing pseudoparticles containing the indicated viral envelopes were treated with IFNα (1,000 IU/mL) for 1 hour, washed thoroughly, incubated for 1 hour, and the extracellular media collected and used to inoculate naïve Huh-7.5 cells (A). The levels of HCVpp-H77 associated E2 were measured on p24-normalized particle preparations by ELISA (B). The effect of IFNα (1,000 IU/mL, 1 hour) on the infectivity of HCVpp expressing a range of genotype 1 glycoproteins (C). HCVpp-H77 were allowed to infect Huh-7.5 cells preincubated for 1 hour with anti-SR-BI (D) or anti-CD81 (E) and infection assessed 72 hours later by measuring luciferase activity. Results are the mean and standard deviation of three assays with duplicate samples where the significance of differences between sensitivities to neutralization at each timepoint was assessed using paired *t* tests (*P < 0.05, **P < 0.01, ***P < 0.005). IFNα had comparable effects on the sensitivity of HCVcc (H77/JFH) to neutralization by anti-SR-BI. Data are presented as IC_50_ values (F).

To ascertain whether HCV particles released from IFNα-treated cells are defective at entering target cells, we used lentiviral pseudoviruses expressing HCV glycoproteins to measure the impact of IFNα on receptor-dependent particle entry. HCVpp (strain H77)-expressing cells were treated with IFNα (1,000 IU/mL) for 1 hour, washed thoroughly, and extracellular media collected to quantify viral infectivity. As controls, we evaluated the effect of IFNα on the infectivity of pseudoviruses expressing Lassa virus and Murine Leukemia virus (MLV) envelope glycoproteins. IFNα reduced the infectivity of HCVpp by 60% but had minimal effect on Lassa or MLVpp infectivity (Fig. 4A). Importantly, IFNα had no detectable effect on the level of HCVpp-incorporated E2 glycoprotein (Fig. 4B), suggesting a similar phenotype to that observed for HCVcc, where intact pseudoparticles are released but are unable to enter cells. To assess the breadth of this phenotype we assessed the impact of IFNα treatment on the infectivity of pseudoparticles expressing a panel of genotype 1 HCV glycoproteins. Of note, five of the 10 envelopes showed a significant reduction in particle infectivity, whereas IFNα had no effect on the infectivity of the remaining pseudoparticles (Fig. 4C). We next investigated the receptor usage of HCVpp released from IFNα-treated or untreated cells. HCVpp-H77 released from IFNα-treated cells showed an increased sensitivity to neutralization by anti-SR-BI, showing an median inhibitory concentration (IC_50_) of 0.06 μg/mL compared to 0.9 μg/mL for untreated virus, but no change to anti-CD81 (Fig. 4D,E), suggesting that virus released from IFN-treated cells had a reduced dependency on SR-BI for entry. We observed a similar reduction in the sensitivity of HCVcc (H77/JFH) released from IFN-treated cells to neutralization by anti-SR-BI (Fig. 4F).

### IFNα Alters HCV E2 Glycoprotein Conformation

**Fig 5:**
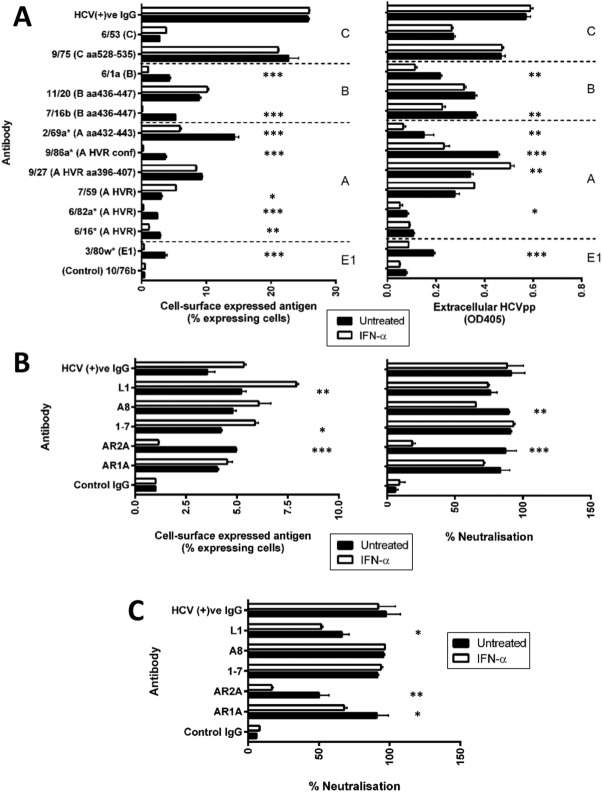
IFNα perturbs HCV glycoprotein conformation. HCVpp-H77 producer 293T cells were treated with IFNα (1,000 IU/mL) for 1 hour, washed thoroughly, incubated for a further hour, and the reactivity of a panel of mAbs targeting linear E1E2 epitopes or a control polyclonal anti-HCV Ig to cell surface expressed antigen or extracellular HCVpp measured by flow cytometry or ELISA, respectively (A). A panel of human antibodies targeting conformation-dependent E2 epitopes (1 μg/mL) were tested for their ability to bind HCVpp-H77-expressing 293T cells or to neutralize extracellular virus following IFNα (1,000 IU/mL, 1 hour) treatment (B). Human antibody anti-E2 (1 μg/mL) neutralization of HCVcc (H77-JFH) released from IFNα (1,000 IU/mL, 1 hour) treated or untreated cells (C). Results are the mean and standard deviation of three experiments and significance was calculated using paired *t* tests (*P < 0.05, **P < 0.01, ***P < 0.005).

Since SR-BI has been reported to bind antigenic region A^13^ of E2 glycoprotein, including the hypervariable region 1 (HVR1), these results suggest that IFN may promote conformational changes within the glycoprotein. To test this hypothesis we evaluated the effect of IFNα on the reactivity of a panel of antibodies targeting linear and conformation-dependent E2 epitopes. Anti-E2 antibodies were assessed for their ability to bind IFNα-treated or untreated HCVpp-producer cells by flow cytometry or to bind extracellular HCVpp by ELISA. IFNα reduced the binding of antibodies targeting HVR1 and region A (6/16, 6/82A, 9/86A, 2/69A), region B (7/16B, 6/1A), but had no significant effect on those binding region C (Fig. 5A). Further studies with a number of human anti-E2 antibodies showed that IFNα reduced the binding and neutralizing activity of antibody AR2A that recognizes a conformation-dependent epitope for HCVpp (Fig. 5B). We confirmed that IFN reduced the sensitivity of HCVcc (H77/JFH) to neutralization by AR2A and had modest effects on AR1A and L1 neutralization (Fig. 5C). Given the low levels of glycoprotein expression at the surface of HCVcc-infected cells, we were unable to evaluate antibody binding to the infected cell surface. The results confirm that IFNα induces a conformational change in the viral E2 glycoprotein that limits particle entry.

## Discussion

We show that IFNα induces a rapid response in HCV-infected hepatoma cells that limits the genesis of infectious extracellular particles with minimal effect on the intracellular viral burden. This short 2-hour duration of IFN treatment significantly reduced *de novo* transmission events, including cell-free and cell-cell dissemination. There was limited evidence for IFN-dependent cellular retention of infectious particles, consistent with a previous report that tetherin, an interferon-stimulated protein, has minimal impact on HCV infectivity.^14^ Previous studies reported that IFNα reduced viral RNA and protein expression in HCV-infected Huh-7 cells or those supporting HCV replicons within 12-16 hours.^4^,^15^ However, our studies focus on the first 2 hours following IFN treatment, highlighting a new role for IFN to limit particle infectivity prior to reducing viral RNA and protein expression. Given the difficulties in measuring the infectivity of primary HCV strains, clinical studies measuring IFN efficacy rely on genomic RNA measurements and may underscore the impact of early IFN doses. A recent model of HCV replication kinetics that measured intracellular RNA and infectivity concluded that the NS5A inhibitor daclatasvir has two modes of action: to block viral RNA synthesis and virion assembly/secretion.^16^ The authors commented that analysis of HCV kinetic data following IFNα treatment with this new model suggested that IFNα reduces HCV assembly/secretion in addition to its role in slowing intracellular HCV RNA replication. Our data are consistent with this interpretation and highlights the importance of studying particle infectivity, i.e., the ratio of particle infectivity to genomic RNA, allowing one to discriminate the effect of drugs on virus assembly, secretion, and infectivity.

Our recently published single-cycle infection assay^12^ enabled us to assess the “early” effects of IFNα on HCV dissemination. By using a short treatment time targeted specifically at infected cells, we demonstrate that IFNα inhibits both cell-free and cell-cell dissemination routes. We confirmed that this effect on particle infectivity and transmission occurred significantly earlier than degradation of viral proteins and is not explained by secreted antiviral factors. Since HCV is a lipid-rich particle that depends on the very low density lipoprotein (VLDL) secretion machinery for assembly and secretion,^17^,^18^ we assessed the density of viral particles released from IFNα-treated cells and saw no obvious changes in particle density. IFNα treatment had no significant impact on the level of viral-associated core or E2 glycoprotein, demonstrating that intact lipoviral particles with reduced infectivity are secreted from IFN-treated cells.

The HCVpp system measures glycoprotein-dependent entry and enabled us to address the role of IFNα on the virus internalization process. IFN treatment of HCVpp 293T producer cells reduced the infectivity of extracellular virus with minimal effect on E2 incorporation into particles. Previous reports have shown that IFNα inhibits HIV-1 particle infectivity by reducing gp120 incorporation into the nascent particles^19^; however, this is unlikely to explain the effect of IFNα on HCVpp infectivity. IFNα had no detectable effect on the infectivity of pseudoviruses expressing MLV or Lassa envelopes, suggesting that this effect is specific to HCV. It is interesting to note the varying effect of IFNα on the infectivity of pseudoparticles expressing a panel of genotype 1 patient-derived clones. HCVpp and HCVcc released from IFNα-treated cells showed an increased sensitivity to the neutralizing activity of an antibody targeting the cellular receptor SR-BI but not CD81, suggesting that IFNα modulates E2 glycoprotein conformation and this may affect receptor utilization and particle entry. Previous studies have shown that region A-HVR of the viral E2 glycoprotein binds SR-BI^20^–^22^; we therefore used a panel of mAbs targeting linear and conformational E2 epitopes to study the effect of IFNα on glycoprotein conformation. We noted significant differences in the ability of several mAbs recognizing linear and a conformation-dependent epitope (AR2A) to bind viral glycoproteins or to neutralize virus infectivity following IFNα treatment.

This phenotype is comparable to reports showing that treatment of HCV-infected cells with glycosidase inhibitors led to the incorporation of misfolded glycoproteins into nascent particles.^23^,^24^ However, glycosidase inhibitors reduced the infectivity of intracellular and extracellular HCV, while IFN only reduced extracellular virus infectivity, suggesting that HCV glycoproteins undergo a late maturation step that is required for maximal particle infectivity.^25^,^26^ Maturation of HIV-1 glycoproteins has been shown to “mask” neutralizing epitopes behind a glycan shield,^27^,^28^ and recent studies suggest a similar model for HCV. Glycosylation plays an important role in defining E2 conformation, with up to eight glycan sites reported to be critical for protein folding.^29^ We failed to detect any significant change in the mobility of virion associated E2 from IFN-treated virus by SDS-PAGE or sensitivity to glycosidase inhibitors (data not shown), suggesting that IFNα-mediated effects on HCV E2 conformation are not glycan-specific.

The results shown here have a particular significance as therapies move to IFN-free regimens of DAAs such as protease and polymerase inhibitors. The ability of IFNα to prevent cell-free and cell-to-cell spread may contribute to the efficacy of IFN in responsive patients. Entry inhibitors have long been suggested as useful adjunct therapies to current treatments, as they would significantly impair virus dissemination and aid the clearance of infected cells. It is possible that short treatments with IFNα may continue to play a significant role in patients treated with DAAs, by preventing viral dissemination.

## Abbreviations

DAA: direct-acting antiviral
HCV: hepatitis C virus
HCVcc: HCV cell-culture virus
HCVpp: HCV pseudoparticle
IFNα: interferon-alpha
ISG: interferon-stimulated gene
mAbs: monoclonal antibodies
